# Analytic Non-adiabatic Couplings for Selected Configuration Interaction via Approximate Degenerate Coupled Perturbed Hartree–Fock

**DOI:** 10.1021/acs.jctc.3c00601

**Published:** 2023-11-08

**Authors:** Jeremy P. Coe

**Affiliations:** Institute of Chemical Sciences, School of Engineering and Physical Sciences, Heriot-Watt University, Edinburgh EH14 4AS, U.K.

## Abstract

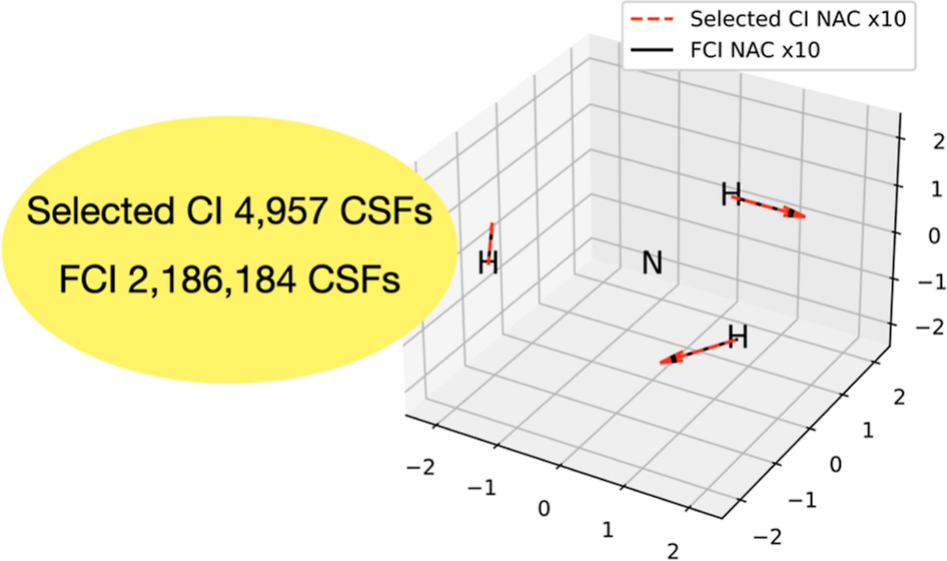

We use degenerate perturbation theory and assume that for degenerate pairs of orbitals, the coupled perturbed Hartree–Fock coefficients are symmetric in the degenerate basis to show  is the only modification needed in the original molecular orbital basis. This enables us to develop efficient and accurate analytic nonadiabatic couplings between electronic states for selected configuration interactions (CIs). Even when the states belong to different irreducible representations, degenerate orbital pairs cannot be excluded by symmetry. For various excited states of carbon monoxide and trigonal planar ammonia, we benchmark the method against the full CI and find it to be accurate. We create a semi-numerical approach and use it to show that the analytic approach is correct even when a high-symmetry structure is distorted to break symmetry so that near degeneracies in orbitals occur. For a range of geometries of trigonal planar ammonia, we find that the analytic non-adiabatic couplings for selected CI can achieve sufficient accuracy using a small fraction of the full CI space.

## 1 Introduction

The non-adiabatic coupling (NAC) between electronic states arises when multiple Born–Oppenheimer potential energy surfaces are important; see, e.g., ref (1) for a review of the theory and applications. Therefore, they can be essential when simulating photochemistry, for example, and components of the NAC are used to locate conical intersections^2−5^ or analyze Jahn–Teller effects (e.g., ref (6)). For efficiency and accuracy, it is crucial to calculate NACs from the electronic structure method using analytic derivatives^7^ rather than numerical derivatives.

Selected configuration interaction (CI) iteratively builds up the electronic wave function by adding and removing configurations based on criteria for their importance. It does not require an active space to be chosen, so it removes the requirement of expertise and the possibility of bias. Selected CI initially used perturbation estimates of a configuration’s significance,^8^ and recently, there has been much successful research into going beyond this approach, which includes refs (9)–^28^. Those works have demonstrated that selected CI can be particularly useful for multireference problems, where small corrections to a single determinant can perform poorly. These multireference situations can encompass bond breaking/forming, molecules containing transition metals, and excited states. Selected CI has been shown to model these systems well using a very small fraction of the full CI (FCI) space. Selected CI therefore has the potential to be of great use for quantum dynamics if analytic NACs can be created for the method.

Unlike orbital-optimized approaches or CISD which has an invariant subset of orbitals, selected CI is not necessarily invariant with rotations between any pair of orbitals. The challenge with analytic NACs for selected CI is that all pairs of orbitals potentially have to be considered for the standard coupled perturbed Hartree–Fock (CPHF)^29^ equations. For pairs of orbitals that are either both occupied or unoccupied in the HF wave function, then these equations contain , where ϵ_*c*_ is the energy of orbital *c*. Hence, these terms become infinite when there are degeneracies. Previously, for analytic gradients for selected CI, we showed^30^ that orbital pairs belonging to different irreducible representations (irreps) do not contribute and found for a range of molecules that the sets of degenerate orbitals split into different irreps when the largest abelian subgroup is used. However, selected CI analytic NACs can be between states of different irreps, and therefore, orbital pairs of different irreps cannot necessarily be excluded. Hence, in this work, we use degenerate perturbation theory with the approximation that for degenerate pairs of orbitals, the coupled perturbed Hartree–Fock coefficients are symmetric in the degenerate basis to show that the only change required is that CPHF coefficients for degenerate pairs are  rather than infinite in the original molecular orbital basis. This allows us to construct analytic NACs for selected CI between general states, regardless of which irreps they belong to, and demonstrate that they are accurate despite using a small fraction of the FCI space.

For state-averaged multiconfiguration self-consistent field (SA-MCSCF) followed by CISD, often termed MR-CI, analytic NACs were created by Lengsfield, Saxe, and Yarkony in ref (7). Later work includes the development of new approaches for MCSCF^31^ and MR-CI.^32^ Analytic NACs have then been created for state-averaged complete active space self-consistent field (SA-CASSCF) with density fitting.^33^ CASCI with floating occupation numbers had analytic NACs implemented in ref (34), and SA-CASSCF analytic NACs using graphical processing units have been developed.^35^ Analytic NACs have also recently been created for spin–orbit MRCI,^36^ dynamically weighted CASSCF,^37^ and SA-CASSCF with the polarizable continuum model (PCM).^38^ Multistate CASPT2 has had analytic NACs developed,^39^ as has CASPT2 with the ionization potential–electron affinity shift.^40^

Analytic NACs have been created for CIS,^41^ spin-flip CIS,^42^ and time-dependent density-functional theory with the conductor-like PCM.^43^ Coupled cluster approaches have had analytic NACs derived and implemented.^44−47^ An efficient method of calculating overlaps between wave functions has been created that can be used for fully numerical NACs,^48^ and artificial neural networks have also been trained to predict NACs as part of surface hopping molecular dynamics.^49^ Approximate analytic NACs have been developed for the state-averaged density matrix renormalization group self-consistent field approach,^50^ and the orbital-optimized variational quantum eigensolver of hybrid classical/quantum computation has also had analytic NACs created.^51^

To develop analytic NACs for selected CI, we first show that, with the assumption that for degenerate pairs of orbitals, the coupled perturbed Hartree–Fock coefficients are symmetric in the degenerate basis, the only changes to the CPHF coefficients in the original molecular orbital basis are  for degenerate pairs of orbitals. We then discuss the approach of ref (7) for NACs of SA-MCSCF wave functions, and we create a seminumerical NAC method for selected CI. Next, we present how we adapt the framework of analytic gradients for selected CI^30^ to efficiently analyze NACs for selected CI and include the contributions from degenerate pairs of orbitals. The analytic NACs for selected CI are then benchmarked against those of FCI singlets using full-space SA-MCSCF in MOLPRO.^52^ As the latter approach optimizes all orbitals, it limits the comparison of NACs to small basis sets. We demonstrate that our NAC approach can reproduce FCI results for carbon monoxide using STO-3G and that the accuracy for selected CI NACs is good when reasonable cutoffs are used, including in the vicinity of an avoided crossing. However, with larger cutoffs, there could be discrepancies when the states are very close in energy, although the results are qualitatively correct. We then look at NACs between states belonging to different irreps of trigonal planar ammonia and again show that the FCI result can be reached. For states where degenerate pairs of orbitals can contribute to the selected CI NAC due to symmetry, we find that including this contribution makes the selected CI NAC slightly more accurate compared with FCI, but we point out that this is not guaranteed and the NAC is not variational. We then increase the basis set to 6-31G, and as the FCI NAC using SA-MCSCF does not use symmetry, it requires about 9 million Slater determinants. Here, we see a behavior similar to that for the smaller basis, but we note that the interstate coupling used for finding and characterizing conical intersections shows a larger percentage error when degenerate pairs of orbitals are neglected. We then break the symmetry of trigonal planar NH_3_ using shifts in coordinates of 10^–3^ bohr and show that the analytic NAC for a larger cutoff selected CI wave function is verified by the seminumerical approach as the step size is lowered. Finally, we vary the bond length of trigonal planar ammonia and consider the NAC between the first two states of A_1_. For large cutoffs, there are some points of discrepancy attributed to states becoming very close in energy or crossing, although the results are, in general, qualitatively correct. For reasonable cutoffs, we find, for this system, that selected CI can give accurate NACs using a small fraction of the FCI space.

## 2 Theory

### 2.1 Degenerate Coupled Perturbed Hartree–Fock

Degenerate perturbation theory overcomes the problem of infinities of standard perturbation theory when the original zero-order eigenfunctions  have the same energy (see, e.g., ref (53)). It does so by using the fact that these eigenfunctions in a degenerate set can be rotated among themselves while remaining zero-order eigenfunctions. They are thus chosen to also be eigenfunctions of the perturbation matrix *H*′ in the subspace of the degenerate set. We term these eigenfunctions the degenerate basis . Hence, this *H*′ is diagonal in the subset of the new degenerate orbitals (but has no reason to be diagonal in general), and this means that in the equations for standard first-order perturbation
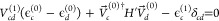
1

 is zero when *c* and *d* are orbitals from the same degenerate set and ϵ_*c*_^(0)^ = ϵ_*d*_^(0)^. Therefore, rather than having infinite values for *U*_*cd*_^(1)^, the *V*_*cd*_^(1)^ values are now not defined by the first-order perturbation equations. Here, we have used the notation of Gerratt and Mills,^29^ where *U*_*in*_ is the *i*’th entry in the MO vector .

In CPHF, the perturbation is^29^
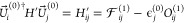
2where  is the first-order correction to the Fock matrix, and *O*_*ij*_^(1)^ is the first-order correction to the overlap, both in the original real, orthonormal MO basis, where the  are unit vectors. The perturbation equations (eq 1) also do not determine the *U*_*jj*_^(1)^, where CPHF are found^29^ from the orthonormalization condition *U*_*ij*_^(1)*^ + *U*_*ji*_^(1)^ + *O*_*ji*_^(1)^ = 0. For *i* = *j* and real orbitals, this gives . For a pair from a degenerate set, we have . Although *H*′ for CPHF (eq 2) is not generally symmetric due to the ϵ_*j*_^(0)^ term, but for a degenerate set, ϵ_*c*_^(0)^ equals ϵ_*d*_^(0)^, so *H*′ is symmetric in this subspace.

We can get an expression for *V*_*cd*_^(1)^ from the second-order CPHF equations^29^ by expressing  as a sum over  then acting on the left with  and using that *H*′ is diagonal in the degenerate orbital subspace to give
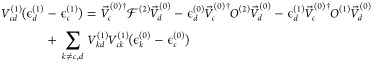
3

For NACs, we are interested in *V*_*cd*_^(1)^ when we cannot use symmetry arguments to exclude its contribution because the change in geometry alters the point group. This means that there is no symmetry reason for ϵ_*d*_^(1)^ = ϵ_*c*_^(1),^ and the equation should give *V*_*cd*_^(1)^. However, the expression contains second derivatives, so we approximate *V*_*cd*_^(1)^ by assuming it is symmetric. This approximation has been used for *U*_*cd*_^(1)^ in earlier work on analytic gradients, e.g., refs (54) and (55) where the antisymmetric part of *U*^*X*_*A*_^ could be set to zero for degenerate orbitals. From the orthonormalization condition, we then have
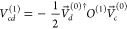
4for degenerate pairs of orbitals *c* and *d*. However, the post-HF calculation is not done in the  basis but in the original MO basis , so next, we look at expressing these in terms of the new basis.

### 2.2 Change to Degenerate Basis

As *H*′ in CPHF (eq 2) is symmetric for a degenerate subset of size *D*, then it may be diagonalized in this space to give real orthonormal eigenfunctions . If we order the orbitals so the first *D* are degenerate, then the transformation to this new set of degenerate orbitals is then effected by  and . As the eigenfunctions are orthonormal, then *P* is an orthogonal matrix, and we have *P^†^P* = *I* and *PP*^†^ = *I*, which in terms of matrix elements is
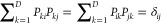
5

### 2.3 Non-degenerate Molecular Orbitals

In CPHF, the  in the basis of *M* molecular orbitals (MOs) are unit vectors, so we can write the first-order perturbation equation for a non-degenerate MO as
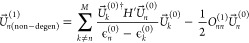
6

If we assume there is only one set of degeneracies, and use the degenerate basis, then we have
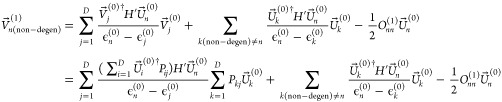
7

As ϵ_*j*_^(0)^ = ϵ_*D*_^(0)^ is constant within the degenerate set, and using eq 5 as *P* is an orthogonal matrix we have
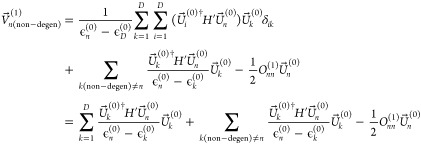
8which is the same as eq 6 hence .

If we have a second set of degenerate orbitals of size *D*_2_, then we order the orbitals so the first degenerate set is 1 to *D*, and the second is *D* + 1 to *D + D*_2_. We now also have the orthogonal matrix *Q* formed from the eigenfunctions of *H*′ in the second degenerate set and . Hence,  now has the additional term
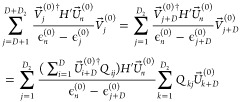
9

Using that  is constant within the second degenerate set and that *Q* is an orthogonal matrix, so  means this is
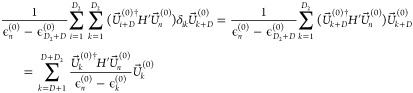
10which again is the same as if the original MO basis had been used. Further sets of degenerate orbitals will just add additional terms in this manner, so we have that  in general.

### 2.4 Degenerate Molecular Orbitals

Continuing with two sets of degenerate MOs, for the first set of degenerate MOs, we have
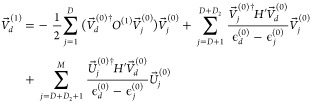
11where the first sum is over the first degenerate set, the second sum is over the second degenerate set, and the final term is summed over the nondegenerate orbitals. As each sum has an odd number of orthogonal matrices, we can see that this will be different from the standard . However,  is the change to , but we want to know the change in the original MOs , hence we transform back to the original MO basis using *P^†^*, so .

The final term summing over the nondegenerate orbitals in  is then
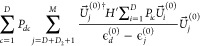
12using the fact that *P* is orthogonal gives
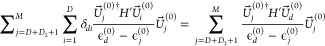
13which is the same as nondegenerate perturbation theory.

For the term over the second set of degenerate orbitals in , we have
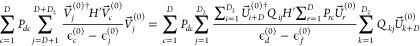
14where we have used that ϵ_*j*+*D*_^(0)^ is constant in the sum over the second degenerate set and written it as ϵ_*f*_^(0)^. While ϵ_*c*_^(0)^ is constant in the sum over *c* (the first degenerate set), it is made equal to ϵ_*d*_^(0)^ Then, we use the fact that *P* is an orthogonal matrix, as is *Q*, and sum over *c* and *j* to give
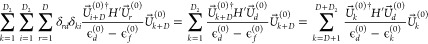
15which again is the same as in standard perturbation theory. If there are further sets of degenerate orbitals, then there is just a term similar to the one above for each one, so this holds in general.

Finally, we have the summation term over the first set of degenerate orbitals, i.e., those that are degenerate with orbital *d*
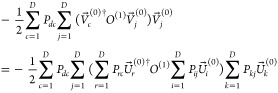
16

As *P* is an orthogonal matrix, we sum over *c* and *j* to give
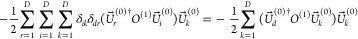
17

So the only change to the standard CPHF result is that the previously infinite *U*_*cd*_^(1)^ when *c* and *d* are degenerate orbitals becomes . In the notation used for selected CI analytic gradients,^30^ when the perturbation is a change in nuclear geometry, this is
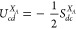
18where *c* and *d* are degenerate.

This result is equivalent to an approximation used in previous works on analytic gradients, e.g., refs (54) and (55), where the antisymmetric part of *U*^*X*_*A*_^ could be set to zero for degenerate orbitals.

#### 2.4.1 Numerical Demonstration

As an example of the use of the degenerate CPHF result, we consider the selected CI analytic gradient of carbon monoxide using STO-3G with two frozen orbitals. We use a bond length of 2.1316 bohr and orientate the molecule as OC. We initially use symmetry for the calculation of RHF MOs, where in *C*_2*v*_ there are two pairs of degenerate orbitals that separate into different irreps. However, we then mix these degenerate orbitals to remove symmetry by rotating the lower-energy degenerate pair by 35° and the other degenerate pair by 70°. This means that we cannot use the symmetry arguments of ref (30) for *F*_*cd*_, the CI Lagrangian, to be zero to deal with degenerate pairs for selected CI analytic gradients. We then run a selected CI calculation with a reasonably large cutoff by using Monte Carlo CI (MCCI)^56,57^ with *c*_min_ = 5 × 10^–3^. We find that Δ*F*_*cd*_ is indeed now nonzero with a magnitude of around 10^–3^ for the degenerate pairs, and as this is used as  in the calculation of the analytic gradient, then the  values for degenerate pairs must also be calculated.

The *U*^*X*_*A*_^ values for nondegenerate orbitals are found by solving the CPHF equations^29^ which are derived from the perturbation equation
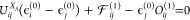
19and in the notation of selected CI analytic gradients^30^ are for *i* an unoccupied RHF MO and *j* an occupied RHF MO
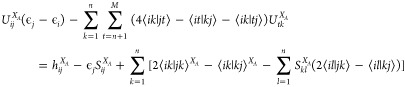
20

When *p* and *q* are either both occupied or unoccupied RHF MOs but nondegenerate, the *U*^*X*_*A*_^ are found from
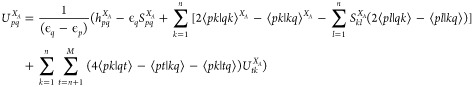
21

**Figure 1:**
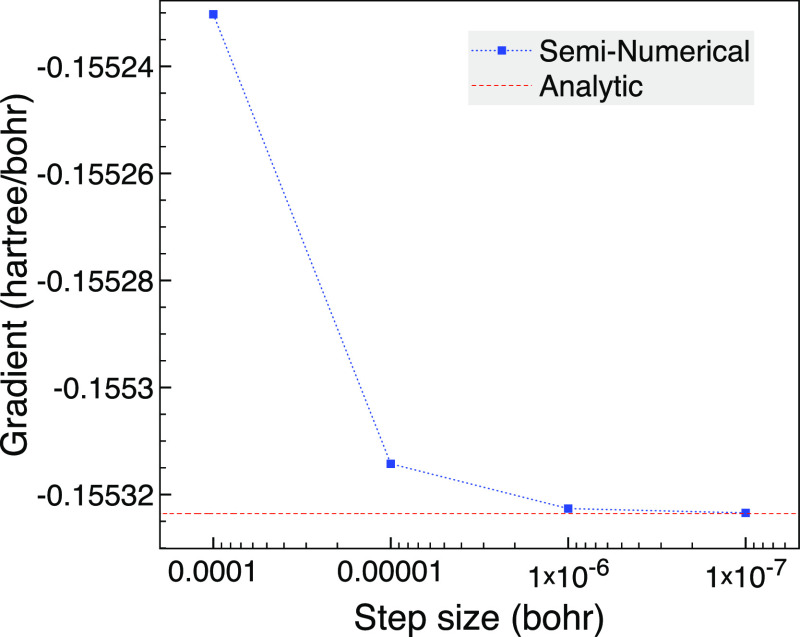
Analytic and seminumerical selected CI gradients for carbon monoxide with STO-3G when degenerate orbital pairs are rotated to remove symmetry using a bond length of 2.1316 bohr, 2 frozen orbitals, and MCCI with a cutoff of 5 × 10^–3^.

Using  for the degenerate pairs, we find that these values are zero, although they are not zero in general, as we shall see later in the NAC calculations. The use of  from degenerate CPHF with the assumption that *V*_*cd*_^(1)^ is symmetric actually gives the exact selected CI analytic gradient result in this case when we mix the degenerate orbitals to remove symmetry, as shown in Figure 1, where we verify the analytic result by showing that the seminumerical calculation converges to it.

### 2.5 NACs

We briefly summarize the approach of Lengsfield, Saxe, and Yarkony in ref (7), where the NAC is divided into
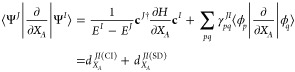
22Here
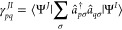
23is the one-particle transition reduced density matrix (transition 1RDM). The first term in eq 22 is called the CI contribution in ref (7). and contains the ***h***^*JI*^ term used as part of searching for and analyzing conical intersections^2−5^
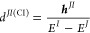
24where it is termed the interstate coupling vector. The second term in eq 22 is denoted as the CSF contribution in ref (7), but as we are using SDs for the selected CI NAC calculations, we refer to it as the SD contribution.

### 2.6 Semi-Numerical

We first use this to create a semi-numerical approach where only a single selected CI calculation is required, but extra RHF calculations are necessary. As only a single selected CI calculation is used, accuracy issues that may occur in fully numerically selected CI derivatives, such as a change in included orbitals between geometries, are avoided. The CI term is
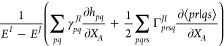
25and the numerical derivative of the MO integrals is calculated using forward differences and a step size of *h* for the shift in geometry, with an extra RHF calculation and then an AO to MO calculation for each degree of freedom. Here
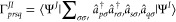
26is the two-particle transition reduced density matrix (transition 2RDM), and
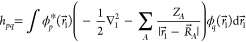
27while
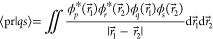
28

The SD term is also approximated by forward differences with step size *h*
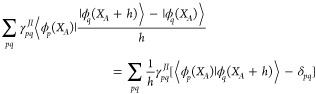
29where the overlap of the MOs at different geometries needs to be calculated, and we enforce phase and order consistency of MOs between different geometries.

For general NACs, the semi-numerical approach needs RHF calculations, then AO to MO transformations of *O*(*M*^5^) for each of the 3*N*_A_ degrees of freedom, so it is not efficient and is used here to verify the analytic approach when there are near degeneracies. We note that when there are degeneracies due to symmetry, the semi-numerical approach will not be applicable if the shift in geometry alters the point group and causes the MOs to change abruptly.

### 2.7 Analytic Non-adiabatic Coupling

#### 2.7.1 CI Contribution

We calculate the CI contribution by adapting the analytic gradient of the electronic energy for selected CI^30^ from  to . So instead of spin free reduced density matrices, the spin free transition-reduced density matrices γ^*JI*^ and Γ^*JI*^ are used. These are computed by adapting the approach^58^ for the 2RDM (Γ = Γ^*JJ*^) of selected CI wave functions. As the excited states in the selected CI use the same MOs, we just have different SD coefficients for the bra than for the ket when calculating the transition 2RDMs.

There is a loss of permutational symmetry, however, compared with γ_*pq*_ and Γ_*prsq*_ as now we only have Γ_*prsq*_^*JI*^ = Γ_*rpqs*_^*JI*^ when *J* ≠ *I*. Hence, one has to be careful if this has been exploited in the implementation. Working through the derivation of analytic gradients leads to γ_*pq*_ being replaced with  and Γ_*prsq*_ with  when contracting with integrals in the code. For example, *F*_*tp*_, sometimes termed the CI Lagrangian, becomes
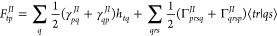
30

While *F*_*tp*_ for analytically selected CI gradients is zero when *t* and *p* are from different irreps,^30^ this is not necessarily the case for *F*_*tp*_^*JI*^, as *J* and *I* can also be from different irreps, so this has no reason to be zero by symmetry if, for example, the irrep of *J* and *t* are the same, while the irrep of *I* and *p* are also the same. *F*_*tp*_^*JI*^ is then used as  in the analytic NAC, where Δ*F*_*pq*_^*JI*^ = *F*_*pq*_^*JI*^ – *F*_*qp*_^*JI*^ and calculated efficiently using the *Z* vector approach.^59^ In the *Z* vector approach, we do not include the degenerate pairs, and they are incorporated as an additional term in the analytic NAC of .

We currently back-transform the transition 2RDMs for efficiency at a cost scaling of *O*(*M*^5^) for each one rather than transform the two-electron AO derivative integrals to the MO basis at a cost of *O*(3*N*_A_*M*^5^). However, we note that if we require NACs for *N*_S_ states, there are now  transition 2RDMs, so when this is greater than 3*N*_A_ it will become more efficient to not back transform the transition 2RDMs.

#### 2.7.2 SD Contribution

The SD term can be written as^7^
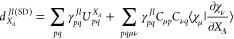
31

The second term is straightforward using the AO integrals, and the back transform of γ_*pq*_^*JI*^ is used
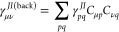
32

The first term is calculated using the approach to give the contribution of a change in the MO coefficients to the analytic gradient. As this is , then we replace *F* by 0.5γ^*JI*^ and use the *Z* vector approach, not including degenerate pairs. They are accounted for with the separate term .

For states *J* and *I*, we run two *Z* vector calculations: one for the CI part and one for the SD part. However, as they avoid the *O*(*M*^5^) scaling of transforming the two-electron derivative integrals, this part is not a bottleneck. We can reduce this to a single *Z* vector calculation for each pair of states if we combine *F*^*JI*^ and γ^*JI*^ into , although this means we cannot separate out the interstate coupling term from eq 24.

#### 2.7.3 Implementation

The atomic orbital (AO) integrals and their derivatives are calculated using the Libcint library^60^ via PySCF.^61,62^ This includes the AO overlaps between different geometries used for the seminumerical NAC approach. PySCF is also used to give the molecular orbital coefficients and energies for restricted Hartree–Fock (RHF).

The selected CI wave functions are computed using the state-averaged MCCI approach^63^ (SA-MCCI), which is based on the MCCI method and code.^56,57^ MCCI adds configurations randomly and uses the cutoff value *c*_min_ to control the accuracy and size of the calculation, as configurations are eventually removed if their absolute coefficient in the resulting wave function is less than this. As the cutoff tends to zero, the FCI wave function is approached. SA-MCCI combines the magnitudes of a configuration’s coefficients for different states when determining whether it should be removed. The RHF molecular orbitals are used for all states, so to account for the change in MOs due to a variation in geometry, only the CPHF equations need to be solved. We note that although we use MCCI in this paper, the NAC approach created here can work with any selected CI method, with the only stipulation being that the RHF molecular orbitals are used for all states.

Configuration state functions (CSFs) are used in the selected CI calculations so that we have pure-spin states. We adapt the efficient 2RDM method of ref (58) to calculate the transition 2RDMs (eq 26), from which the transition 1RDMs are found. There, the CSFs are transformed to SDs using the method of Löwdin^64^ in the calculation of the 2RDMs.

The selected CI analytic gradients method from ref (30) is then built upon for NACs and includes the contribution from degenerate pairs of molecular orbitals.

## 3 Results

### 3.1 Carbon Monoxide

We first verify the method and use of PySCF integrals by comparing them with FCI NAC results for carbon monoxide using a different program: MOLPRO^52,65,66^ full-space SA-MCSCF calculations. As these are costly and require that no orbitals be frozen for NACs, this limits these comparator FCI NAC results to small basis sets. For this system, we do not need to consider NACs between states of different irreps; if we write the NAC using forward differences , then for a diatomic, a change in bond length cannot change the irrep, so this is zero by symmetry when *J* and *I* belong to different irreps.

**Figure 2:**
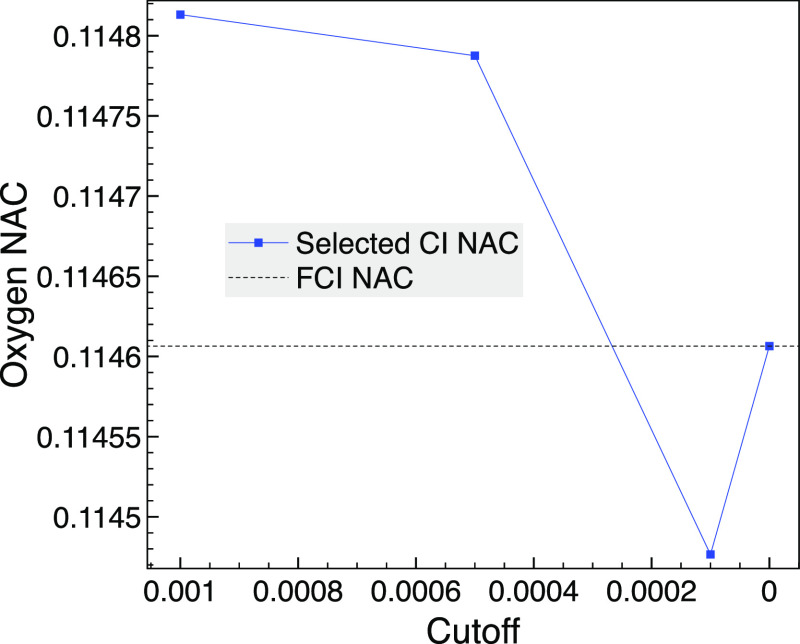
Comparison of selected CI NACs, as the cutoff is varied for SA-MCCI, and the FCI NAC between 1A_1_ and 3A_1_ for the oxygen of CO with a bond length of 3 bohr using the STO-3G basis set and *C*_2*v*_.

A bond length of 3 bohr is used with the STO-3G basis and the largest abelian subgroup of *C*_2*v*_. Although there are two sets of doubly degenerate MOs that split into different irreps when using *C*_2*v*_, they will not have any effect as we are limited to NACs between states of the same irrep. In the A_1_ irrep and looking at singlets, the first nonzero NAC at this geometry is between the ground state (state 1) and state 3. The NAC of the oxygen atom is plotted in Figure 2, with the wave function phases chosen to be consistent. There we see that the selected CI NACs are accurate and approach the FCI result as the MCCI cutoff is lowered. At a cutoff of zero, the FCI wave function is reached, and the selected CI NAC result agrees to 8 decimal places with the MOLPRO full-space SA-MCSCF result.

**Figure 3:**
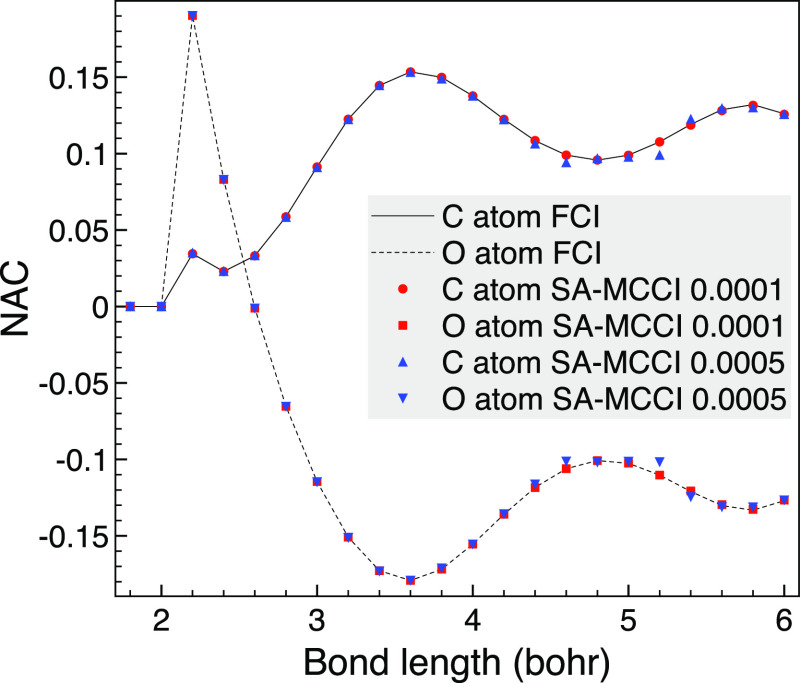
Comparison of selected CI using SA-MCCI and FCI NACs between 1A_1_ and 3A_1_ of CO as the bond length is varied when using the STO-3G basis set and the *C*_2*v*_ point group.

When varying the bond length, we see in Figure 3 that with a cutoff of 10^–4^ for MCCI, the NACs are essentially indistinguishable from FCI. With a larger cutoff of 5 × 10^–4^, there is some discrepancy as the molecule approaches dissociation. We attribute this to the system becoming more multireferenced and the states starting to coalesce, so the  factor in the CI part of the NAC will be large and amplify errors in the selected CI calculation. For example, at *R* = 5.6 bohr and a cutoff of 10^–3^, we find 0.103 for the NAC on carbon, of which 0.107 is the CI part.

We quantify the accuracy with FCI using the root-mean-square error (RMSE) shown in eq 33. Here, *N*_A_ is the number of atoms, and *N*_g_ is the number of geometries included in the potential energy curve.
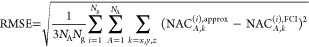
33

Table 1 shows that the accuracy of the selected CI NAC values is high once the cutoff is below 10^–3^. However, due to the small FCI space of 1378 CSFs, the selected CI approach does not have a good opportunity to demonstrate that it only needs a tiny fraction of the configurations for these benchmark calculations. We will look at larger FCI spaces in the next section.

**Table 1:** Errors Using RMSE When Compared with FCI for the NACs between the 1A_1_ and 3A_1_ States of CO, and the Mean Number of CSFs across the 22 Geometries When Using the STO-3G Basis Set

method	NAC error	mean CSFs
MCCI 1 × 10^–^^3^	1.32 × 10^–^^2^	184
MCCI 5 × 10^–^^4^	0.136 × 10^–^^2^	227
MCCI 2 × 10^–^^4^	0.100 × 10^–^^2^	285
MCCI 1 × 10^–^^4^	0.0124 × 10^–^^2^	337

The main source of error is at points when the energy curves are close in energy as the molecule approaches dissociation. If we do not include bond lengths greater than 5 bohr, then we see in Table 2 that the accuracy increases by around an order of magnitude.

**Table 2:** Errors Using RMSE When Compared with FCI for the NACs between the First and Third A_1_ States of CO and Mean Number of CSFs across the 17 Geometries ≤5 bohr When Using STO-3G

method	NAC error
MCCI 1 × 10^–^^3^	0.278 × 10^–^^2^
MCCI 5 × 10^–^^4^	0.0786 × 10^–^^2^
MCCI 2 × 10^–^^4^	0.0334 × 10^–^^2^
MCCI 1 × 10^–^^4^	0.00347 × 10^–^^2^

**Figure 4:**
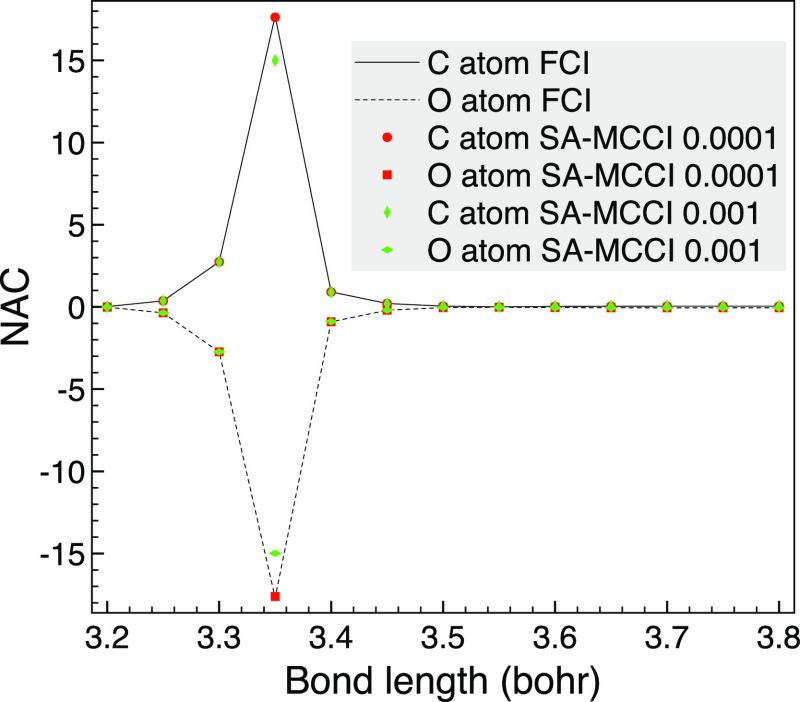
Comparison of selected CI and FCI NACs between 2B_1_ and 3B_1_ of CO as the bond length is varied in the vicinity of an avoided crossing when the STO-3G basis set and *C*_2*v*_.

We next look at NACs between B_1_ singlet states of CO, as the potential energy curves of these states have avoided crossings that were investigated in ref (67). We see in Figure 4 that the selected CI NACs with a cutoff of 10^–4^ are essentially FCI quality, while even with a large cutoff of 10^–3^ the shape is correct, and although the absolute error at the avoided crossing may be large, its magnitude has been captured.

We see in Table 3 that, due to the large NAC around the avoided crossing, the errors are larger than for the A_1_ results (Table 1). However, the accuracy is sufficiently high for a cutoff of 5 × 10^–4^ or less, and the cutoff of 1 × 10^–3^ gave qualitatively correct NACs, as we saw in Figure 4. Although the small basis set means that a large proportion of the 1220 CSFs from the FCI space have been used.

**Table 3:** Errors Using RMSE When Compared with FCI for the NACs between the Second and Third B_1_ States of CO and Mean Number of CSFs across the 13 Geometries When Using STO-3G

method	NAC error	mean CSFs
MCCI 1 × 10^–^^3^	0.433	224
MCCI 5 × 10^–^^4^	5.72 × 10^–^^2^	268
MCCI 2 × 10^–^^4^	3.84 × 10^–^^2^	320
MCCI 1 × 10^–^^4^	1.26 × 10^–^^2^	373

We next go beyond a diatomic and look at NH_3_ so that we can investigate selected CI for nonzero NACs between states of different irreps.

### 3.2 Ammonia

**Figure 5:**
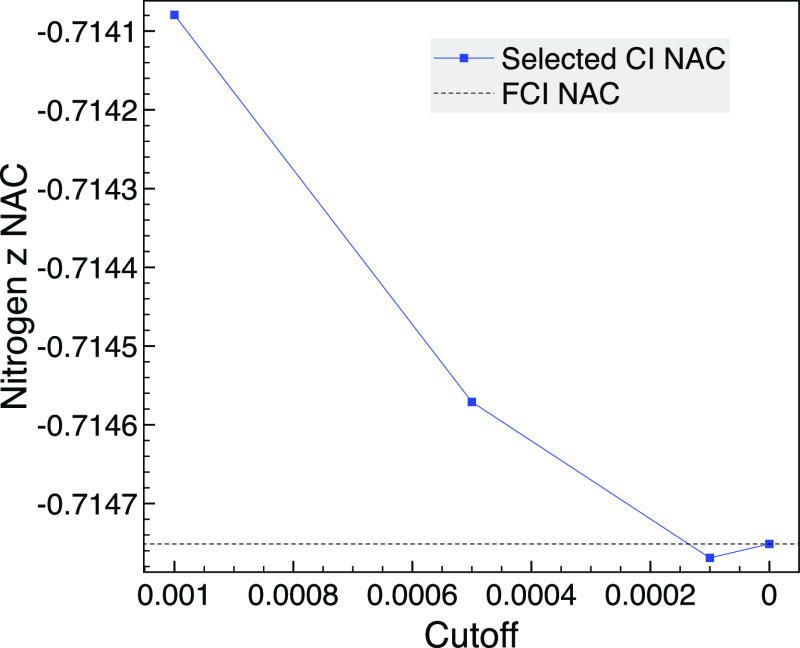
Comparison of the selected CI *z* component of the NAC between 1A_1_ and 1B_2_ for the nitrogen of trigonal planar NH_3_ using MCCI with various cutoffs and the corresponding FCI calculation without symmetry at a bond length of 1.2 Å and using the STO-3G basis set.

We first demonstrate that the approach works when the results of two selected CI calculations of different irreps. They are compared with FCI via full-space SA-MCSCF from MOLPRO,^52^ which does not allow NACs between different irreps, so symmetry cannot be used in the SA-MCSCF calculation. For singlet states of trigonal planar NH_3_ at a bond length of 1.2 Å and using the STO-3G basis set and *C*_2*v*_, we display the convergence of selected CI NACs between 1A_1_ and 1B_2_ in Figure 5. The full-space SA-MCSCF without symmetry used 1176 CSFs, and the two states of interest are the first two states when symmetry is not used. The selected CI calculations with symmetry ranged from 102 A_1_ and 87 B_2_ CSFs for a cutoff of 10^–3^ to 155 and 138 CSFs for 10^–4^. The FCI result using the selected CI with a cutoff of zero agrees with the MOLPRO FCI result to 8 decimal places. Although there are two sets of doubly degenerate orbitals, they will not contribute to the selected CI calculation as they are A_1_ and B_1_ pairs.

We next look at a B_1_ state with the ground A_1_, where the contribution of degenerate orbitals is expected to be nonzero. The ground B_1_ state is degenerate with the third A_1_ state, which means an FCI calculation using SA-MCSCF without symmetry has the possibility of mixing states. Hence, for a robust benchmark, we look at the 2B_1_ state, which is the eighth state at this geometry when symmetry is not used.

**Figure 6:**
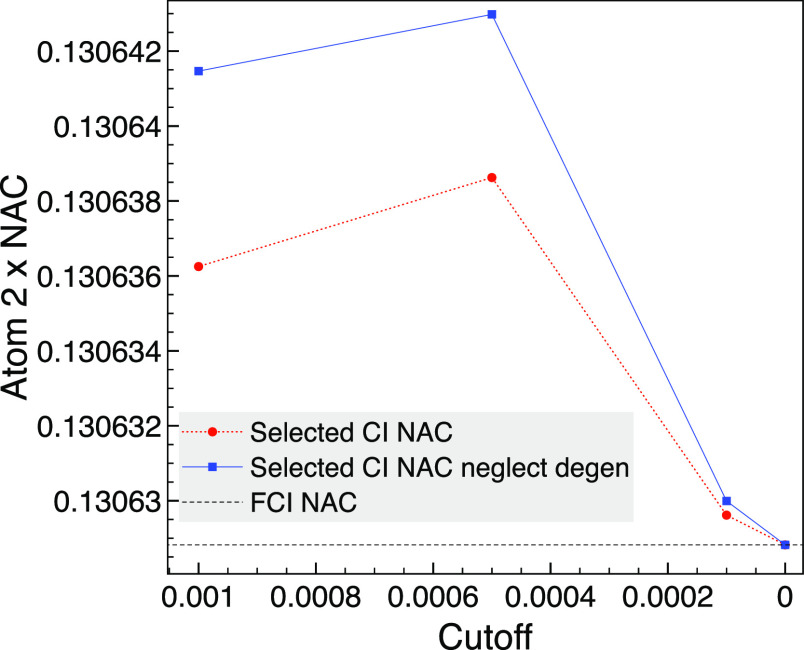
Comparison of the selected CI NAC with the result when neglecting degenerate MO pairs (neglect degen) for the *x* component of the NAC between 1A_1_ and 2B_1_ for atom 2 (hydrogen) of trigonal planar NH_3_ using MCCI with various cutoffs and the corresponding FCI calculation without symmetry at a bond length of 1.2 Å and using the STO-3G basis set.

The results are shown in Figure 6 where we see that all selected CI results capture the FCI NAC to four decimal places, and the FCI result is reproduced by the *c*_min_ = 0 calculation to 8 decimal places. We compare the results by neglecting the contribution of degenerate MO pairs by setting their  values to zero. By not excluding pairs of degenerate MOs, the selected CI result is closer to FCI for nonzero cutoffs on the scale of the graph, but the NAC is not variational, and the absolute change is very small.

**Figure 7:**
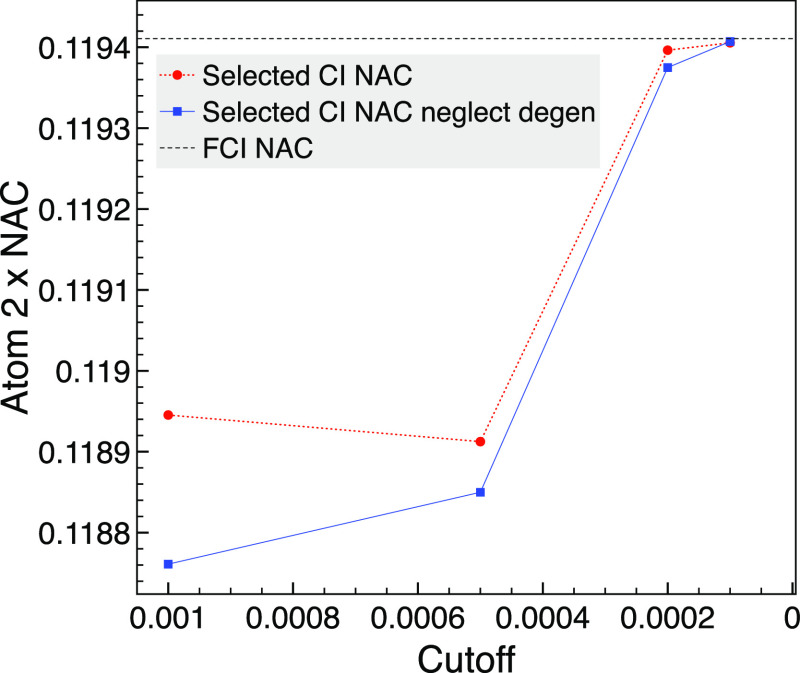
Comparison of selected CI NAC with the result when neglecting degenerate MO pairs (neglect degen) for the *x* component of the NAC between 1A_1_ and 2B_1_ for atom 2 (hydrogen) of trigonal planar NH_3_ using MCCI with various cutoffs and the corresponding FCI calculation without symmetry at a bond length of 1.2 Å and using the 6-31G basis set.

We now increase the basis to 6-31G, which gives 4 sets of doubly degenerate orbitals for this geometry. The FCI NAC calculation using full-space SA-MCSCF does not employ symmetry, so it is reasonably challenging with 2,186,184 CSFs (9,018,009 determinants). At this geometry, the eighth state still corresponds to 2B_1_ with this basis and is not degenerate. With the larger basis, we see in Figure 7 that the selected CI results all give the FCI NAC to three decimal places rather than four, as was the case with the smaller basis. The selected CI wave functions that combined the 1A_1_ and 2B_1_ MCCI results ranged from 2245 to 18,529 CSFs. Again, the neglect of degenerate pairs does not make much difference, particularly as the cutoff is lowered. These NAC values appear closer to the FCI result when degenerate orbitals are accounted for, but this is not the case if we consider the y NAC of atom 3, which gets a little further from the FCI result at a cutoff of 2 × 10^–4^ (0.011% error if neglecting degenerate compared with 0.018%).

**Figure 8:**
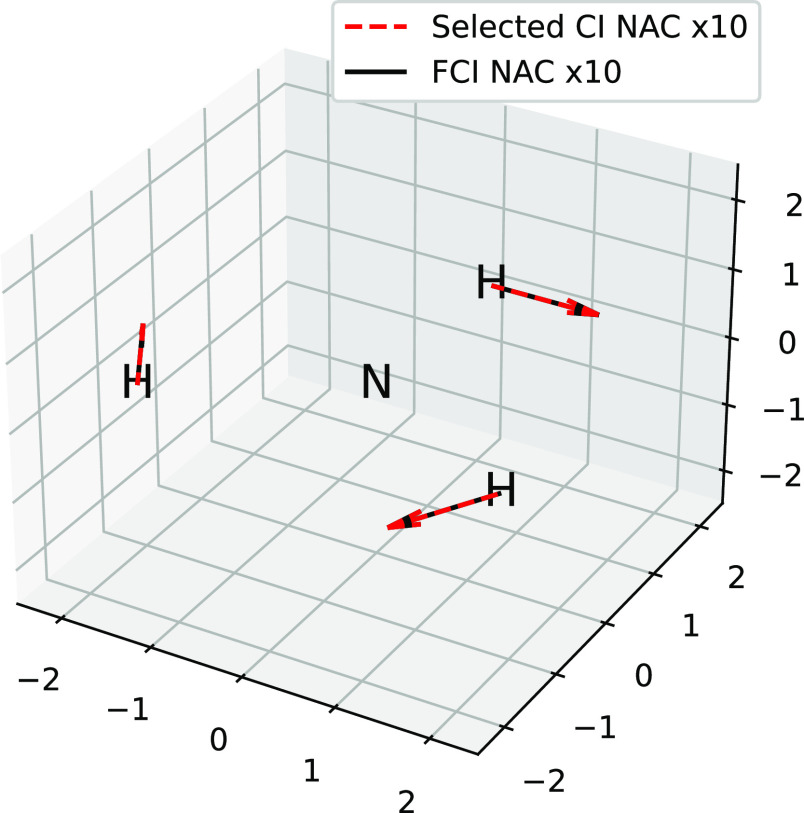
Comparison of selected CI NACs between 1A_1_ and 2B_1_ and the corresponding results of FCI all scaled by a factor of 10 for trigonal planar NH_3_ using MCCI with a cutoff of 5 × 10^–4^ at a bond length of 1.2 Å and the 6-31G basis set.

We also plot the NACs for all atoms and coordinates in Figure 8, where we scale them by a factor of 10. We see that even with this scaling, the selected CI NACs with a cutoff of 5 × 10^–4^ are indistinguishable from FCI on the scale of the graph.

**Figure 9:**
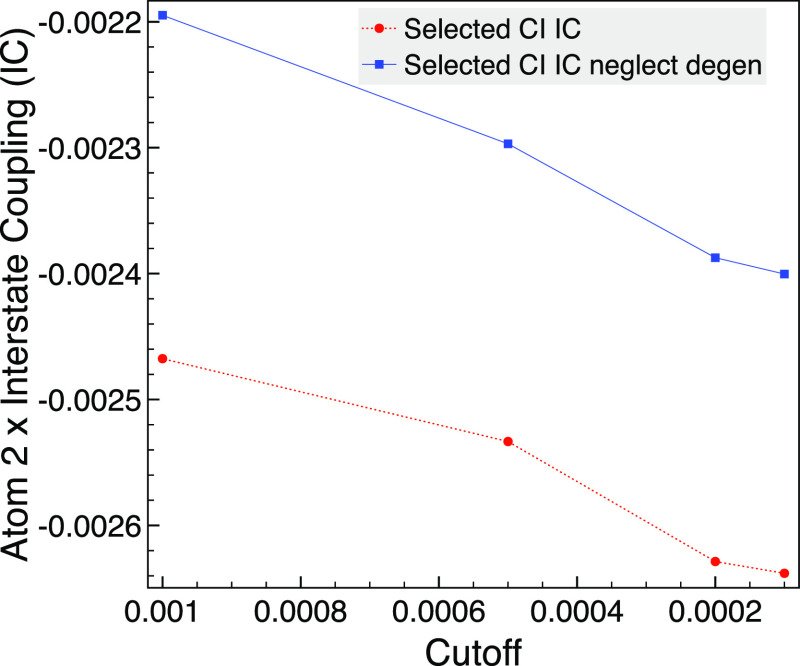
Comparison of selected CI NAC with neglecting degenerate MO pairs (neglect degen) for the *x* component of the interstate coupling (IC) between 1A_1_ and 2B_1_ for atom 2 (hydrogen) of trigonal planar NH_3_ using MCCI with various cutoffs, a bond length of 1.2 Å, and the 6-31G basis set.

The interstate coupling from eq 24 is also considered for this system using the selected CI in Figure 9. There, we see that the effect of neglecting degenerate orbitals is more important, as the error can be around 10%, although the values are smaller than the NACs. As the interstate coupling is used as part of the analysis of conical intersections^2−4^, then we speculate that including degenerate orbital pairs could become important if selected CI is employed in this context. Figure 9 suggests that the interstate coupling would also be affected by neglecting degenerate pairs in the FCI limit. This is because Δ*F*^*JI*^ is not necessarily zero for invariant orbital spaces, as was shown in ref (32) for MRCI NACs; however, they demonstrated that its contributions instead canceled those due to Δγ^*JI*^ for invariant orbital spaces.

**Figure 10:**
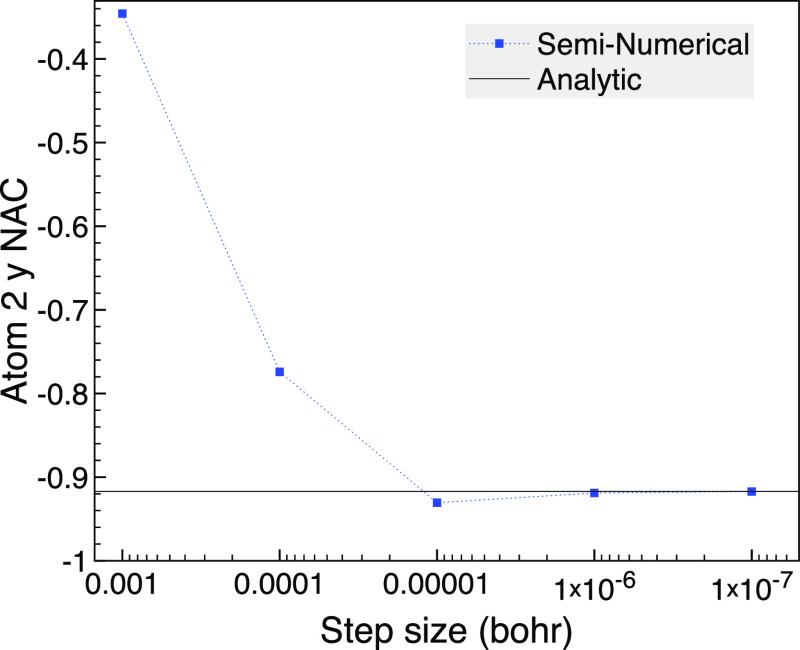
Comparison of seminumerical and analytic selected CI *y* NAC of atom 2 between the first two singlet states for broken symmetry NH_3_ using 10^–3^ bohr changes based on the trigonal planar geometry at a bond length of 1.5 bohr using 6-31G and MCCI with a cutoff of 10^–3^.

We next look at the challenge of near degeneracies by breaking the symmetry of trigonal planar NH_3_ in 6-31G by shifting the *x* and *z* coordinates of the nitrogen by 10^–3^. We use MCCI with a 10^–3^ cutoff to give 907 CSFs and validate the NAC using the seminumerical approach rather than comparing with FCI. For the first two states, the *y* value of the NAC for atom 2 is challenging, as can be seen in Figure 10, where the seminumerical approach is quite different from the analytic value until the step size is 10^–5^ bohr or less. The analytic NAC has to contend with near degeneracies as low as 4 × 10^–6^ that are accompanied by Δ*F*^*JI*^ values that can be around 10^–5^. This suggests their contribution cannot be neglected, yet they are not close enough to be classified as degenerate. In fact, if we treat orbital pairs with energy differences less than 10^–5^ as degenerate, we get a noticeably different value of −0.29 for the NAC; however, using a threshold of 10^–6^ instead, we get the analytic result in Figure 10 that is verified by the seminumerical approach. Due to the states not being very close at this bond length, the NAC is not dominated by the CI contribution (−1.25) as there is a non-negligible SD part of 0.33.

**Figure 11:**
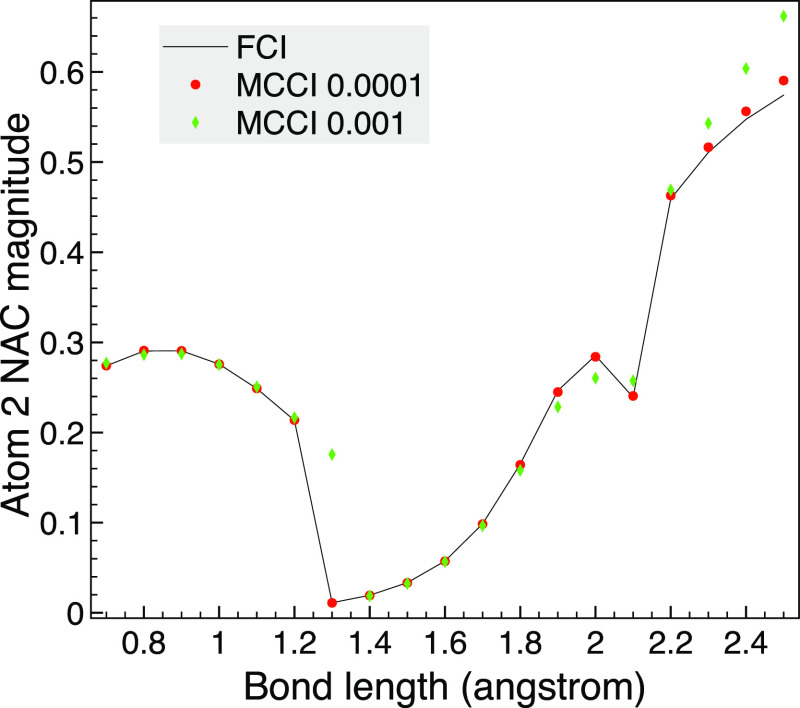
Comparison of selected CI and FCI NAC magnitudes for atom 2 (hydrogen) between singlet states 1A_1_ and 2A_1_ of trigonal planar NH_3_ as the bond length is varied when the 6-31G basis set is used and *C*_2*v*_.

We now look at varying the bond lengths of trigonal planar NH_3_ and calculate the NACs between the first two states of A_1_ when using 6-31G. The FCI benchmark using SA-MCSCF for all orbitals now has 561,322 CSFs (2,261,477 determinants) when using A_1_. We see in Figure 11 that with a cutoff of 10^–4^ the selected CI NAC magnitudes are FCI quality except for a slight difference when the bonds are very stretched and the energies for the two states become close, meaning that small errors in the energies are exacerbated due to the  factor in the NACs. With a cutoff of 10^–3^, the accuracy is lower, but the FCI curve is qualitatively captured except for a point at 1.3 Å not being zero, which we link to the excited state potential energy curve having a noticeably higher energy at this cutoff. There, it appears it may correspond to the third FCI state, which is close in energy to the second state at this geometry. Furthermore, the second and third states are very close around 1.2 Å and may cross, which links in with the sudden drop in the NAC around this point. The few points of discrepancy when using a cutoff of 10^–3^ could perhaps be rectified by using the previous geometry’s wave function as a starting point, but they are definitely removed by going to the lower cutoff.

We see in Table 4 that the larger FCI space makes the NAC curve more challenging than the smaller basis set of CO examples. However, this also allows selected CI to use a much smaller fraction of the FCI space, and a cutoff of 2 × 10^–4^ gives good accuracy using around 1.3% of the FCI space.

**Table 4:** Selected CI Errors Using RMSE When Compared with FCI for NACs between the 1A_1_ and 2A_1_ States of Trigonal Planar NH_3_ and the Mean Number of CSFs across 19 Geometries When Using 6-31G

method	NAC error	mean CSFs
MCCI 1 × 10^–^^3^	2.00 × 10^–^^2^	1743
MCCI 5 × 10^–^^4^	1.48 × 10^–^^2^	3396
MCCI 2 × 10^–^^4^	0.358 × 10^–^^2^	7354
MCCI 1 × 10^–^^4^	0.250 × 10^–^^2^	12,028

## 4 Conclusions

We have developed analytic non-adiabatic couplings (NACs) for selected CI wave functions by building on the approach for analytic selected CI gradients.^30^ We used degenerate perturbation theory and assumed that for degenerate pairs of orbitals, the coupled perturbed Hartree–Fock coefficients are symmetric in the degenerate basis to show that  was the only modification needed in the original molecular orbital basis. This enabled us to calculate general NACs for selected CI, including between states of different irreducible representations (irreps), where symmetry arguments cannot be used to neglect pairs of degenerate orbitals and standard CPHF would give infinite values.

The method was benchmarked against FCI NACs using full-space SA-MCSCF in MOLPRO.^52^ The SA-MCSCF NAC results were reproduced when using selected CI to perform FCI by including all configurations. We used the selected CI approach of state-averaged Monte Carlo configuration interaction with CSFs, although we emphasize that the NAC procedure can work with any selected CI method.

For carbon monoxide, we found that the NAC between the ground and third singlet states of A_1_ was accurately calculated using selected CI at a bond length of 3 bohr and STO-3G. When varying the bond length, the selected CI NACs were generally accurate, although for larger selected CI cutoffs, there was some discrepancy as the molecule approached dissociation and the states became very close in energy. The NAC between the second and third states of B_1_ was then considered, as this avoided crossing with large NAC values. This meant errors were not as low, but accuracy was high enough when using reasonable cutoffs, and for larger cutoffs, the NACs were still qualitatively correct.

Next, we looked at trigonal planar ammonia, where NACs between states from different irreps could not be excluded due to symmetry, but there are pairs of degenerate orbitals due to symmetry. We first showed that FCI results using a minimal basis could be approached by the selected NAC between 1A_1_ and 1B_2_ when combining the selected CI wave functions of the two irreps. However, the irreps of the degenerate orbital pairs meant they did not contribute to these NACs. Hence, we considered the NAC between 1A_1_ and 2B_1_, where the latter was the first B_1_ state that was nondegenerate, so the FCI benchmark without using symmetry would be robust. We found that neglecting the degenerate pairs slightly reduced the accuracy of the component of the NAC investigated, but all of the selected CI NAC results were of high accuracy. We highlighted that, due to the NAC being nonvariational, including the degenerate orbital pairs is not guaranteed to give a closer result to the FCI. We then used a larger basis set (6-31G), where the FCI space was now around 9 million determinants, and a similar behavior to the smaller basis was observed in the selected CI NACs. The percentage error was higher in the interstate coupling when there were degenerate orbital pairs. As this is used for characterizing conical intersections, we suggested that the inclusion of degenerate pairs may become more important if selected CI is used in this application with high symmetry geometries.

We then broke the symmetry of this system by distorting the geometry of two coordinates by 10^–3^ bohr to test the method with near degeneracies. We used the semi-numerical NAC approach introduced in this paper to verify the selected CI analytic NAC when a large cutoff was used. We found that near degeneracies were as close as 4 × 10^–6^, but as long as they were included in the analytic NAC, the result agreed with the semi-numerical approach when its step size was sufficiently low.

Finally, we varied the bond length for trigonal planar ammonia and calculated the analytic NAC between the first two A_1_ singlet states. The challenge of states becoming close meant that with a large cutoff, there were some geometries with larger errors, but the curve was generally qualitatively correct. While the results were sufficiently accurate with a lower cutoff that used around 7000 CSFs compared with the FCI space of over half a million.

The analytic nonadiabatic couplings between general selected CI wave functions developed in this work will enable future applications of selected CI in the search for conical intersections and for quantum dynamics.
